# Tuning Biocompatibility and Bactericidal Efficacy as a Function of Doping of Gold in ZnO Nanocrystals

**DOI:** 10.1021/acsomega.3c09680

**Published:** 2024-05-10

**Authors:** Jerusa Maria de Oliveira, Davi P. da Silva, Luciana Rosa de S. Floresta, Gustavo G. Rocha, Larissa Iolanda Moreira de Almeida, Edigar Henrique V. Dias, Thaís Karine de Lima, Juliane Z. Marinho, Marylu M. de Lima, Felipe B. Valer, Fábio de Oliveira, Thiago L. Rocha, Valter Alvino, Lucas Anhezini, Anielle Christine A. Silva

**Affiliations:** † Strategic Materials Laboratory, Physics Institute, Federal University of Alagoas, Maceió, CEP: 57072-900 Alagoas, Brazil; ‡ Rede Nordeste de Biotecnologia (RENORBIO), Chemistry Institute, Federal University of Alagoas, Maceió 57072-900, Alagoas, Brazil; § Laboratory of in vivo Toxicity Analysis, Institute of Biological Sciences and Health, Federal University of Alagoas, Maceió 57072-970, Alagoas, Brazil; ∥ Laboratory of Wound Treatment Research, Institute of Pharmaceutical Sciences, Federal University of Alagoas, Maceió 57072-970, Alagoas, Brazil; ⊥ Department of Medicine, Biotechnology Institute, Federal University of Catalão, Catalão 75705-220, Goiás, Brazil; # Institute of Chemistry, Federal University of Uberlândia, Uberlândia 38400-902, Minas Gerais, Brazil; ∇ Laboratory of Environmental Biotechnology and Ecotoxicology, Institute of Tropical Pathology and Public Health, Federal University of Goiás, Goiânia 74605-050, Goiás, Brazil; ○ Department of BioMolecular Sciences, School of Pharmaceutical Sciences of Ribeirão Preto, University of São Paulo, Ribeirão Preto 05508-900, São Paulo, Brazil; ◆ Laboratory of Molecular and Cellular Biology, Institute of Biomedical Sciences, Federal University of Uberlândia, Uberlândia 38408-100, Minas Gerais, Brazil

## Abstract

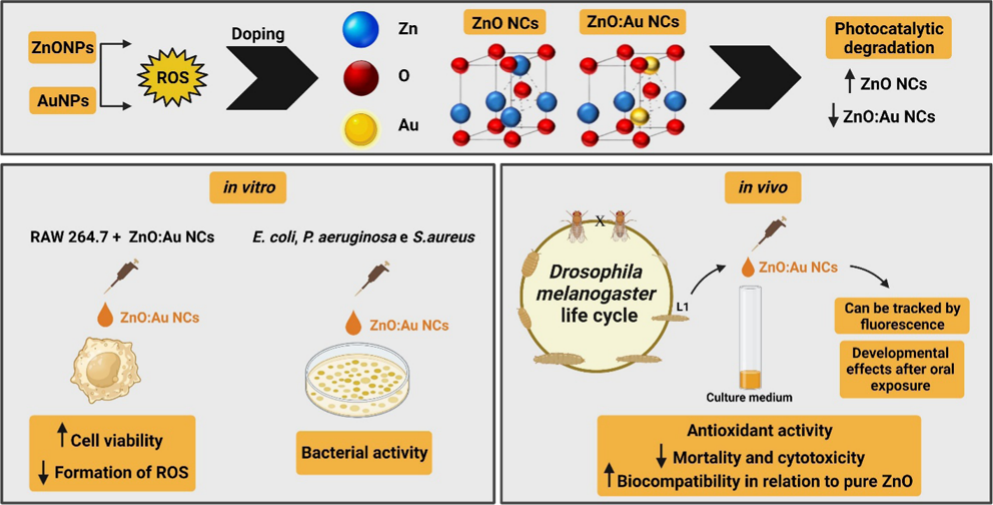

Doping nanoparticles represents a strategy for modulating the energy levels and surface states of nanocrystals (NCs), thereby enhancing their efficiency and mitigating toxicity. Thus, we herein focus on the successful synthesis of pure and gold (Au)-doped zinc oxide (ZnO) nanocrystals (NCs), investigating their physical–chemical properties and evaluating their applicability and toxicity through *in vitro* and *in vivo* assessments. The optical, structural, and photocatalytic characteristics of these NCs were scrutinized by using optical absorption (OA), X-ray diffraction (XRD), and methylene blue degradation, respectively. The formation and doping of the NCs were corroborated by the XRD and OA results. While the introduction of Au as a dopant did induce changes in the phase and size of ZnO, a high concentration of Au ions in ZnO led to a reduction in their photocatalytic activity. This demonstrated a restricted antibacterial efficacy against *Escherichia coli*, *Pseudomonas aeruginosa*, and *Staphylococcus aureus*. Remarkably, Au-doped counterparts exhibited enhanced biocompatibility in comparison to ZnO, as evidenced in both *in vitro* (murine macrophage cells) and *in vivo* (*Drosophila melanogaster*) studies. Furthermore, confocal microscopy images showed a high luminescence of Au-doped ZnO NCs *in vivo*. Thus, this study underscores the potential of Au doping of ZnO NCs as a promising technique to enhance material properties and increase biocompatibility.

## 1 Introduction

The use of nanoparticles (NPs) in manufacturing various products is expanding due to their economically and medically valuable properties. Nanomaterials possess bactericidal action, act as drug nanocarriers, and are utilized in producing sunscreens and other everyday items. NPs are particles ranging from 1 to 100 nm and exhibit distinct properties compared to larger-scale particles of the same material.^1^ Additionally, their large surface area exposes a more significant number of atoms, increasing accessibility and reactivity and leading to greater multifunctionality.^2^

Zinc oxide (ZnO) NPs are commonly used in the manufacturing of cosmetics, food preservatives, paints, fungicides, and plastics. These NPs possess excellent chemical stability, high photostability, and vigorous bactericidal activity.^3^ Metal NPs also showed many different properties, such as gold (Au) NPs exhibiting optical, fluorescent, and magnetic properties, which are particularly interesting for applications in biomedicine. Nonetheless, it is crucial to recognize the potential toxicity of these NPs. Consequently, numerous studies have been undertaken to rigorously assess their biocompatibility and suitability for various applications. Understanding the safety profile of these NPs is fundamental to their responsible and informed utilization in diverse contexts.^4^

ZnO and Au NPs are extensively studied as promising antimicrobial agents, performing antibacterial activities,^5^ antifungal properties,^6,7^ antiparasitic effects,^8,9^ and antiviral potential.^10,11^ They have diverse applications for human and animal health and even agriculture. From this perspective, these NPs have high biotechnological potential, stimulating investigations of their application in developing nanoproducts with biomedical applications.^12,13^

Despite their wide range of applications, these NPs can induce toxicity, both *in vitro* and *in vivo*, through genotoxic effects,^14−17^ the induction of aberrant phenotypes,^18^ liver and kidney toxicity, mitochondrial depolarization, and cell death.^18,19^ Virtually all nanoparticles, including Au NPs^20,21^ and ZnO NPs,^22^ share a common mechanism of toxicity: excessive generation of reactive oxygen species (ROS) and/or nitrogen (RNS) and inhibition of antioxidant enzyme activity with the induction of oxidative stress.^22−26^ The doping of ZnO NPs with transition metals is presented as an alternative to improve their biocompatibility and maintain or enhance their physicochemical properties. These methodologies have proven to be a valuable tool for improving their biomedical properties and their biocompatibility.^27^

The research platform involving nanoproducts based on ZnO–Au nanoparticles is aimed at harnessing the synergy of these nanomaterials to enhance antimicrobial effects and improve biocompatibility performance, making them potential candidates for treating infections caused by multidrug-resistant microorganisms. The antimicrobial activity of ZnO-based nanomaterials is related to their physicochemical properties that lead to irreparable cellular damage.^28^ However, the advantage of using combinations of ZnO and Au NPs as promising antimicrobial agents is linked to the improvement in their efficiency and biosafety, justifying broad application in the biomedical field.

Investigating new nanoproducts and their potential toxicity necessitates a thorough examination, including *in vivo* studies, to assess possible adverse effects of nanomaterial exposure. In this context, the fruit fly (*Drosophila melanogaster*) has been indicated as a suitable model system in nanotoxicological studies due to its various characteristics, such as a well-marked life cycle, which allows all stages of the animal’s life to be easily analyzed in a short period, in addition to having a low maintenance cost.^29^ Also, fruit flies have 77% of conserved genes related to human diseases^30^ and similarities with humans in different physiological mechanisms.^31^ Thus, these characteristics make *D. melanogaster* a great model for biocompatibility studies^29,32−35^ such as biocompatibility assessment of nanomaterials.^29,36^ In addition, *D. melanogaster* is also considered an animal model that aligns with the concept of the three Rs (replacement, reduction, and refinement) as recommended by the European Center for the Validation of Alternative Methods (ECVAM).^37^

In this study, we synthesized pure and Au-doped ZnO nanocrystals (NCs), exploring their potential applications as bactericides. Subsequently, we assessed their biocompatibility using the fruit fly (*D. melanogaster*) as an *in vivo* model. Our hypothesis posited that Au doping would augment the nanoparticles’ biocompatibility and facilitate *in vivo* detection. To investigate this hypothesis, we conducted comprehensive characterization tests on the nanocrystals including *in vitro* cytotoxicity assays and various bactericidal tests. The study aimed to elucidate the impact of gold doping on the nanoparticles’ functionality and their potential as antibacterial agents. Furthermore, we sought to understand the biocompatibility of these Au-doped ZnO NPs in an *in vivo* context, employing fruit flies as a model organism.

As part of our experimental design, we conducted toxicological assessments to evaluate the impact of nanocrystals on the cytotoxicity and mortality of fruit flies during their postembryonic development. This multifaceted approach allowed us to glean insights into both the bactericidal properties and the potential adverse effects on the biological systems, contributing to a comprehensive understanding of the synthesized nanocrystals’ behavior in relevant biological contexts.

## 2 Materials and Methods

### 2.1 Synthesis and Characterization of Nanocrystals

Pure and Au-doped ZnO NCs (ZnO/xAu = 0.5, 1.0, 5.0, 10.0) were synthesized according to the procedures described by Silva et al. as previously described by BR10201800771. All reagents are nearly 99% pure and purchased from Sigma-Aldrich Company. The optical absorption spectra were performed by a UV–vis–NIR spectrometer (Shimadzu). The X-ray diffraction (XRD) measurements were performed on a diffractometer XRD-6000 (Shimadzu) operated at 20 kV and 2 mA with Cu Ka radiation (λ = 1.5406 Å). The XRD diffraction results were refined using Rietveld’s profile analysis method with the General Structure Analysis System (GSAS) program suite with the EXPGUI interface. The hydrodynamic diameter, the zeta potential, and the polydispersity index (PdI) of NCs (3 mg/L) suspended in ultrapure water (Milli-Q) at pH 7.0 were determined using a Malvern ZetaSizer (model Nano-ZS90).^38^ The hydrodynamic diameter and PdI were analyzed by dynamic light scattering (DLS), while the zeta potential was determined by electrophoretic light scattering (ELS).

The photocatalytic activities of samples were tested in methylene blue (MB) degradation under artificial light irradiation with a 300 W xenon lamp as the light source. In detail, 8 mg of photocatalyst was added into a 50 mL MB solution (0.02 mmol/L) in a cylindrical reactor. A magnetic stirrer was used to ensure homogeneous dispersion of the photocatalyst during the reaction. The photocatalytic reactor was positioned 15 cm below the light source, and the photocatalytic reaction was initiated by adding the catalyst into the reactor and switching on the lamp. In controlled intervals, 3.0 mL of suspension was collected for analysis. Adsorption experiments were conducted under the same conditions without xenon light irradiations. MB concentration was measured by a UV–vis–NIR spectrophotometer (Shimadzu) at a wavelength of 663 nm. The formula calculated the percentage of discoloration, % Discoloration = 1 – (*A*/*A*_0_) × 100 (1), where *A* is the absorbance of the solution at time *t* > 0 and *A*_0_ is the initial absorbance.

### 2.2 *In Vitro* Bioassay

#### 2.2.1 Cellular Culture

The RAW 264.7 murine macrophage cells were cultivated in Roswell Park Memorial Institute medium (RPMI 1640 Gibco, Invitrogen, Carlsbad) supplemented with 5% fetal bovine serum (FBS), 100 U/mL of penicillin, 100 U/mL of streptomycin, 2 mM of L-glutamine, 3 mM of sodium bicarbonate, and 25 mM of HEPES. The macrophage cells were maintained at 37 °C and a humidified atmosphere of 5% CO_2_.

#### 2.2.2 Cell Viability

The cell viability post-treatment of pure and Au-doped ZnO NCs was assessed using the MTT (3-(4,5-dimethylthiazol-2-yl)-2,5-diphenyltetrazolium bromide) assay. First, 1 × 10^5^ cells/mL density was cultured in a 96-well plate for 24 h at 37 °C and 5% CO_2_. Afterward, the semiconfluent monolayer was exposed to different concentrations (0, 1, 5, 10, 15 μg/mL) of each Au-doped ZnO NC for 24 h in triplicate. Afterward, the supernatant was removed, and 1 mg/mL/well MTT was added, followed by incubation for 1 h at 37 °C. The formazan crystals were dissolved using dimethyl sulfoxide, and the plate was read at 630 nm. The cell viability was expressed as a percentage of the cellular control (cells treated with the RPMI medium only).

#### 2.2.3 Nitric Oxide Production

The nitric oxide (NO) production was determined by nitrite (NO_2_^–^) accumulation in RAW 264.7 cells. The Griess reagent was used for this assay as described by Chi et al. (2003). Briefly, 1 × 10^6^ cells/mL were seeded in 96-well tissue culture plates and incubated for 24 h at 37 °C and 5% CO_2_. So, the cells were exposed to pure and Au-doped ZnO NCs at 1 μg/mL of concentration in triplicate for 24 h at 37 °C and 5% CO_2_. The lipopolysaccharide (1 μg/mL/well) and RPMI medium supplemented with 5% FBS were used as positive and cellular controls, respectively. For the nitrite measurement, 50 μL of culture supernatant was mixed with 50 μL of Greiss reagent (1:1 solution of 0.1% naphthyl ethylenediamine and 1% sulfanilamide) followed by incubation in the dark for 5 min at room temperature. Finally, the absorbance was measured at 630 nm. The calculation of NO_2_^–^ concentration in supernatants of macrophage cells was based on the standard curve of a 2-fold serially diluted NaNO_2_ (200 μM) solution.

### 2.3 Antimicrobial Activity

#### 2.3.1 Antibacterial Assay

For the antimicrobial evaluations, Au NPs were tested against the strains *Staphylococcus aureus* (ATCC 25923), *Pseudomonas aeruginosa* (ATCC 27853), and *Escherichia coli* (ATCC 25922). The antimicrobial activity was determined by broth microdilution methodology according to NCCLS recommendations.^39^ 96-well microplates were filled with 100 μL of Mueller–Hinton broth. Au NPs were suspended in a saline solution containing 2.5% dimethyl sulfoxide (DMSO) with an initial concentration of 16,000 μg/mL, with serial dilution. The microbial inoculum was prepared using the direct suspension technique, with turbidity adjusted to the standard of 0.5 on the McFarland scale (1.5 × 10^8^ cells/mL), with a volume of 5 μL dispensed in each of the wells of the lines, except for the last line intended for sterility control. Negative (saline solution with 2.5% DMSO) and positive (Meropenem with an initial concentration of 1000 μg/mL) controls were included. The plates were incubated in a bacteriological oven at ±36 °C for 18–24 h, after which a volume of 20 μL of 1% 2,3,5-triphenyl tetrazolium chloride (TTC) was added to all wells. The minimum inhibitory concentration (MIC) was determined by the lowest concentration capable of inhibiting bacterial growth in three different wells.

### 2.4 *In Vivo* Bioassay

#### 2.4.1 Drosophila Stocks and Culture

Animals of the *Canton S* lineage of *D. melanogaster* (Diptera, Drosophilidae) were reared on a standard cornmeal media (cornmeal, agar, yeast extract, NaCl, sugar, and propionic acid) under controlled conditions of temperature (25 ± 1 °C), humidity (60–70%), and photoperiod (12:12 h light–dark cycle). The pure ZnO and Au-doped ZnO samples were suspended in ultrapure water. The stock dispersion (10 mg/mL) was used to prepare appropriate concentrations for *in vivo* analyses. The NCs were then dispersed using a 5 W probe sonicator (Elmasonic EASY 60 H, Misonix) for 30 min before use. It is important to emphasize that these NCs can withstand high temperatures.

#### 2.4.2 Developmental Assays

The developmental assay is essential to evaluate if pure and Au-doped ZnO NCs could affect the overall development of *Drosophila* and cause any lethality during each developmental stage. To perform this experiment, approximately 100 males and females were kept in a 6 oz plastic bottle containing a grape juice-based agar cap at the bottom, and the embryo collection was carried out in 8 h intervals when the caps were replaced. After 24 h of the egg collection, embryos developed into larvae, and these newly hatched larvae were then transferred to experimental vials containing 4 mL of a standard cornmeal media added of six different concentrations (0.0,0.25, 0.5, 1.0, 2.0, and 4.0 mg/mL; the final concentration) of ZnO NCs in three different percentages (0.05,1.0, and 5.0) of the Au and standard media as a control. Six (n = 6) replicates containing 35 larvae were set up for each concentration, and the animals were monitored during the following stages of development for about 10 days at 25 °C.

The larvae that reached the pupal stage were recorded daily. The pupation frequency was analyzed daily and compared to that of the control to determine any delay during larval development. The difference between the initial number of larvae transferred to the media and the total number of pupae indicates the level of larval lethality for each experimental concentration.

After hatching, the animals exposed to ZnO:5.0Au were separated into males and females and maintained in vials containing the standard control medium throughout their lives. Every 3 days, the surviving animals were transferred to a new vial, and the number of deaths was quantified. This process was carried out until all animals were dead. In this way, we determined the life expectancy of animals that developed in media containing nanocrystals compared to control animals in standard media. The results were expressed as the percentage of survival as determined by the log-rank test.

#### 2.4.3 Trypan Blue Exclusion Test

The larvae were exposed to the NCs for 3 days, and on the fourth day, the exclusion test was performed. Trypan blue assay was performed for 30 min in a Petri dish containing agarose (0.8%), trypan blue (10%), and sucrose (5%). After this period, the larvae were washed in phosphate-buffered saline (PBS) 1×. For the positive control group, 0.2 mg/mL of the herbicide Imazapyr (C_13_H_15_N_3_O_3_) (Matt Tiririca Imazapyr commercial solution, Kelldrin, 2.0% w/w) was added to the Petri dish. Subsequently, the animals were inserted into previously identified microtubes and anesthetized for 5 min at −20 °C. Then, images of the whole animals were taken in a stereoscopic microscope (Opticam OPTZR) with a coupled capture system (5.3MP optuhd). Ten larvae per group were analyzed, and the final percentage of intestines that showed blue coloration was considered positive for cell death or damage (the bluer, the lower the cell viability).^40^

#### 2.4.6 Tracking and Fluorescence Intensity Analysis of ZnO- and Au-Doped ZnO Nanocrystals In Vivo

The *Drosophila Canton S* lineage larvae and adults were dissected to verify their exposure to nanocrystals (NCs) and to assess the accumulation of NCs in their tissues. *Drosophila* embryos were collected during a 1 h time window and allowed to mature into first-instar larvae for 24 h. These larvae were then exposed to either ZnO or Au-doped ZnO (ZnO:5.0Au) nanocrystals at a concentration of 1 mg/mL. Subsequently, the gut, fat body, ovaries, and brain tissues of both larvae and adult flies were dissected and fixed using a 4% paraformaldehyde solution for 23 min. The presence of NCs within these tissues was analyzed using an SP5 confocal laser microscope (Leica Microsystems) with a 405 nm laser. To evaluate the potential enhancement of *in vivo* luminescence due to Au doping, we undertook the quantification of the existing luminescence intensity. This quantification was carried out through the ROI statistics plugin on the Icy software.^41^ We applied this analysis to different images acquired from the gut tissues of adult animals exposed to both ZnO and Au-doped ZnO (ZnO:5.0Au) nanocrystals.

### 2.5 Statistical Analysis

All statistical analyses were performed using the GraphPad Prism software (version 6.0, GraphPad Software Inc., San Diego, CA). The MIC values obtained for each experimental and standard treatment group were subjected to analysis of variance using one-way ANOVA followed by Tukey’s test for comparison of means (*p*-value < 0.05). For statistical differences in the NO production assay, one-way ANOVA with Bonferroni’s post-test was used. The pupation rate, larval, and pupal lethality data were submitted for analysis of homogeneity of variance with the Shapiro–Wilk test. Parametric data were subjected to analysis of variance (ANOVA) followed by the Tukey test to mean comparison. The significance level of 5% was adopted, and the data are presented as mean ± standard error of the mean (EPM). The linear regression test tested the curves (pupation/day %) for equality between groups.

## 3 Results and Discussion

### 3.1 Characterization of Pure and Au-Doped ZnO Nanocrystals

**Figure 1:**
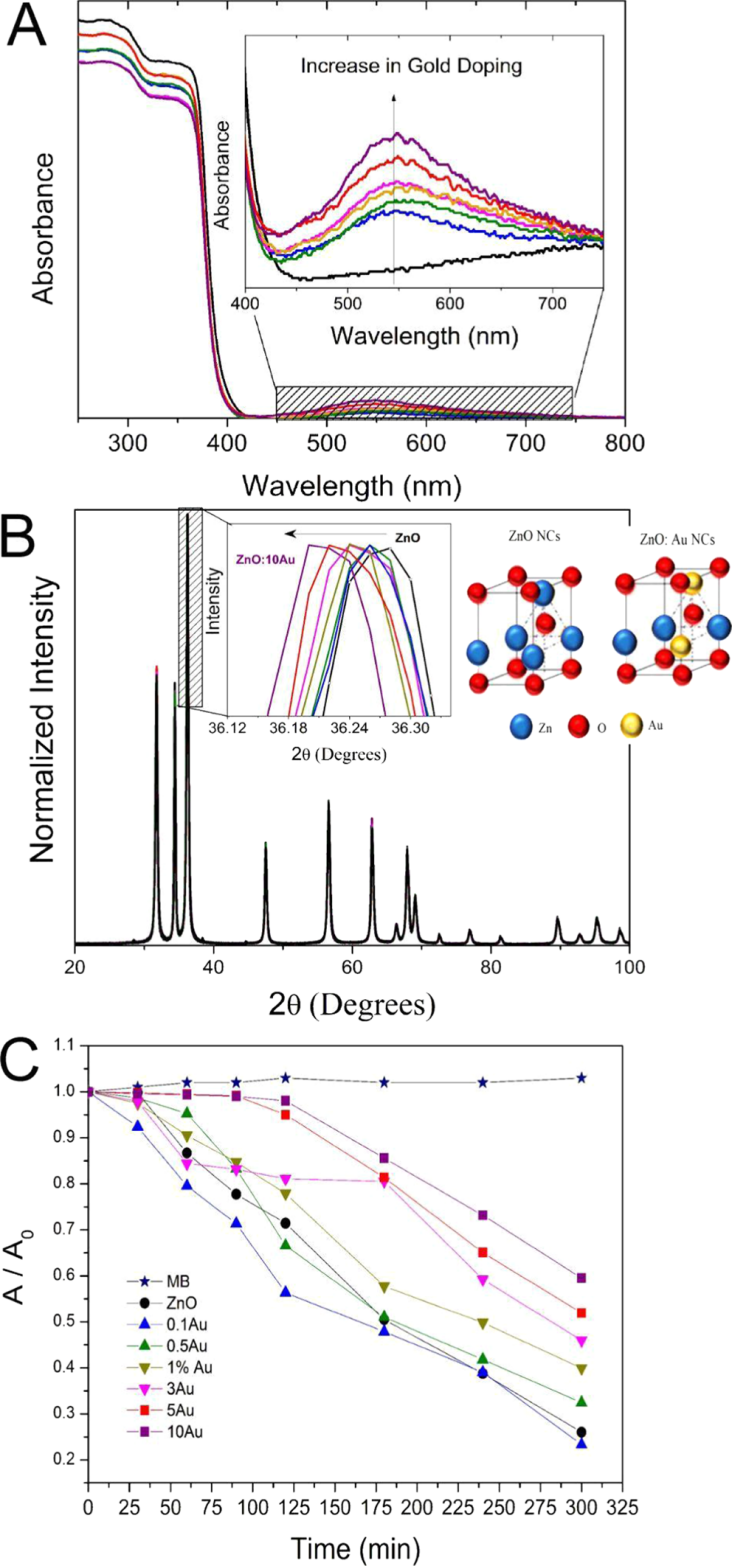
(A) Optical absorption spectra, (B) XRD patterns, and (C) photocatalytic activities of pure and Au-doped ZnO nanocrystals. The insets show the shift of the main peak to smaller angles with increasing Au concentration, and Au^3+^ ions incorporated occupy Zn^2+^ sites in the crystalline structure of ZnO NCs.

The optical and structural properties and photocatalytic activities are shown in Figure 1. The optical absorption spectra of pure and Au-doped ZnO NCs are shown in Figure 1A. A wide band in the 200–400 nm range characteristic of ZnO absorption is observed in the ZnO NC spectrum. In the Au-doped ZnO NCs, a band in the range of 500–700 nm (see the inset) characteristic of Au ions is observed. In addition, in the region close to 450 nm, there was a blue shift with the Au concentration. These results give strong evidence that the Au ions are incorporated into the ZnO nanocrystals.

The XRD diffractograms of samples are shown in Figure 1B, in which the Bragg peaks are characteristics of the standard cards of wurtzite ZnO nanocrystals (JCPDS:36-1451). It is also observed that with increasing concentration of Au, no additional Bragg diffraction peaks are observed. This result indicates that all of the Au ions were possibly incorporated into the ZnO crystal structure. The inset shows the shift of the main peak to smaller angles with an increasing Au concentration, confirming Au-doped ZnO NCs. The Scherrer equation of pure and Au-doped ZnO NCs determines the average size to be around 20 nm.

The XRD patterns refined by the Rietveld method and the results obtained after the final cycle of refinement are shown in Table 1. The cell parameters, a and c, and volume increase subtly with a concentration growth of dopant due to a bigger ionic radius of Au^3+^ (0.68 Å) than that of the Zn^2+^ (0.60 Å), indicating that Au^3+^ ions incorporated occupy the Zn^2+^ sites in the crystalline structure of ZnO, as shown in the inset of Figure 1B.

**Table 1:** Parameters Obtained from the Rietveld Refinement for Pure and Au-Doped ZnO NCs

	cell parameters						
samples	*a* = *b* (Å)	c (Å)	V (Å^3^)	*R*_wp_ (%)	*R*_p_ (%)	*R*_bragg_ (%)	χ^2^
ZnO	3.2504	5.2066	47.640	5.56	3.93	0.80	1.934
0.1 Au	3.2514	5.2077	47.677	5.49	3.56	1.15	1.983
0.5 Au	3.2502	5.2061	47.629	5.82	4.39	0.65	2.245
1.0 Au	3.2513	5.2074	47.673	5.26	3.80	1.03	1.745
3.0 Au	3.2515	5.2081	47.686	4.96	3.64	1.18	1.626
5.0 Au	3.2515	5.2079	47.685	5.43	4.00	1.33	1.954
10.0 Au	3.2515	5.2080	47.686	5.78	4.40	1.44	2.216

The photocatalytic activities of these samples were examined in the degradation of MB aqueous solution under 25 °C with different Au concentrations under UV–vis light irradiation (Figure 1C). The photocatalytic activities of the samples decrease with the Au concentration. After UV–vis irradiation for 5 h, the degradation of MB is found to be 73, 75, 65, 60, 55, 44, and 42% over ZnO NCs and with samples doped with 0.1, 0.5, 1.0, 3.0, 5.0, and 10.0 of Au, respectively. As also observed in the literature, improved photocatalytic properties have been reported for noble metal-modified ZnO nanostructures, such as Au-doped ZnO, Pt-doped ZnO, and Au–Ni-doped ZnO NCs.^41,42^ Efficiency enhancement is often attributed to reduced excitation recombination in ZnO structures by transferring photoexcited electrons to metal nanoparticles.^42^

Anyway, prolonged illumination of the dye solution above the UV–vis light intensity (in the presence of a suitable photocatalyst, such as ZnO) would generate reactive radical species like H^+^ and O_2_^•–^ that mineralizes the dye.^42,43^ In addition, the enhanced photocatalytic activity of ZnO:0.1Au mainly originated from the accelerated generation of O_2_^•–^ during the photodiscoloration process. However, introducing a high level of Au (>0 1) has adverse effects on photocatalytic activity, probably due to the ascending of defect levels. The excess of defects can act as the recombination center of the electron and hole pairs.

The insertion of the metallic nanoparticles in ZnO and hybrid materials resulted in excellent dye degradation activities observed in metal–semiconductor hybrid materials with Pt–Au–ZnO and Au–ZnO hybrids. This can be attributed to the fast discharge of electrons, leading to better electron–hole separation.^42^ Haichuan Mu et al. reported that the superior photocatalytic performance of the graphene/Au/ZnO nanoplates on the Ni foam could be ascribed to the combining effects of the ZnO nanostructure morphology and incorporation of Au NPs.^44^ In the literature, the synergism of Au and ZnO nanoparticles intensifies their photocatalytic properties. However, in this work, Au replaces Zn and interstitial in the ZnO crystalline lattice, causing a decrease in exciton coupling and decreasing photocatalytic properties.

DLS measurements showed similar hydrodynamic diameter and PdI for pure and Au-doped ZnO NCs. DLS results indicated the formation of aggregates/agglomerates of Au-doped ZnO NCs in ultrapure water (1.0 Au = 398.7 ± 38.01 nm; 5.0 Au = 476.7 ± 7.35 nm). Au-doped ZnO NCs were monodisperse in ultrapure water with a PdI of 0.107 ± 0.06 (1.0 Au) and 0.134 ± 0.12 (5.0 Au). In addition, ZnO NC showed a negative zeta potential in ultrapure water (1.0 Au = −13.78 ± 1.73 mV; 5.0Au = −18.68 ± 0.85 mV), indicating the formation of aggregates/agglomerates with a negative surface charge.

### 3.2 Viability and NO Production in RAW 264.7 Macrophages

**Figure 2:**
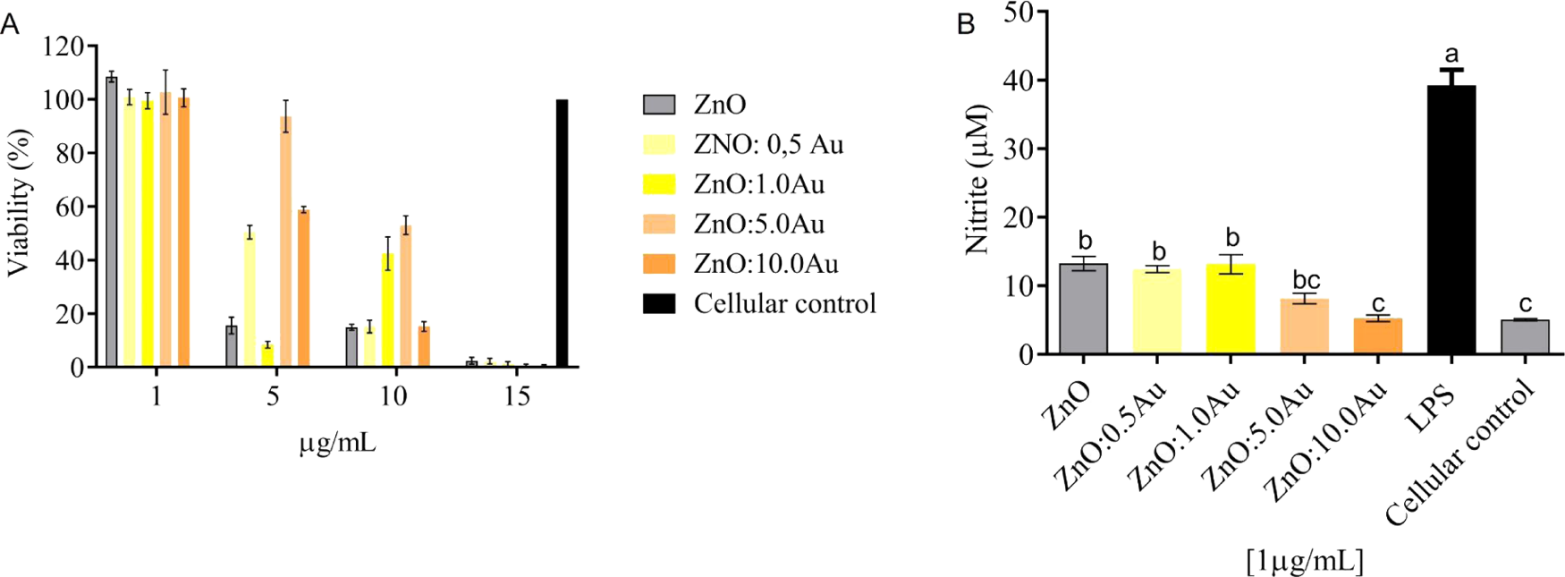
Effect of ZnO nanoparticles and Au-doped ZnO nanocrystals on murine macrophage RAW 264.7 cells. (A) Cell viability in murine macrophage RAW 264.7 cells from the cellular control group and after exposure to different concentrations of pure and Au-doped ZnO NCs. Cell viability was assessed by using the MTT assay. (B) Nitrite production in RAW 264.7 cells after exposure to different concentrations of pure and Au-doped ZnO NCs in murine macrophage RAW 264.7 cells at a 1 μg/mL concentration. For comparisons between concentrations, two-way ANOVA was used. ^a,b,c^ Different letters represent existing differences between concentrations and the control of a nanocrystal.

The viability of RAW 264.7 macrophages after NC treatment is demonstrated in Figure 2. The MTT assay shows that 1 μg/mL of concentration does not influence cell viability, making it the most biocompatible concentration of NCs to use in RAW 264.7 cells after 24 h of incubation. The data reveal a considerable reduction in cell viability after treatment with increasing NC concentrations, starting at 5 μg/mL (viability reduction below 50% in ZnO, 0.5 Au, and 1.0 Au NC treatments). At the highest concentration (15 μg/mL), virtually all cells died. However, cell viability increased when cells were exposed to ZnO:1.0Au and 10.0 Au at a concentration of 5 μg/mL and 1.0 Au and 5.0 Au at a concentration of 10 μg/mL, compared to the ZnO control. As reported in the current study, the concentration of ZnO NCs determines the viability of murine macrophage RAW 264.7 cells.^45^ The mechanism of ZnO NC toxicity can be related to the dissolution of Zn^2+^ ions from the nanoparticles and their release in RAW 264.7 cells, resulting in cell injury through the production of reactive oxygen species (ROS).^46^ These results are consistent with the chemical oxidation results (Figure 2A). Therefore, doping with 5.0 Au was able to reduce the toxic effects of ZnO in RAW 264.7 cells, indicating the improved biocompatibility of ZnO:5.0Au samples at concentrations of 5 and 10 μg/mL.

In this study, oxidative cell injury was measured by quantifying NO^2-^. In Figure 2B, we observed a low production of NO^2-^ by murine macrophage cells when treated with all Au-doped ZnO NCs (*p* < 0.05). Unlike some published studies, our data suggest that NC-related cytotoxicity occurs through mechanisms independent of Zn^2+^ dissolution. This fact can be attributed to the physicochemical properties of ZnO NCs. Particularly in the 5Au and 10Au samples, there was a reduction of 79 and 86% in NO production compared to LPS stimulation, suggesting that higher Au doping concentrations reduce ROS production in this cell type. The reduction in NO^2-^ levels observed in our study may be due to the anti-inflammatory and antioxidant activities of ZnO NCs in macrophages.^47,48^ ZnO NCs also decreased the expression of inflammatory agents such as cyclooxygenase 2 (prostaglandin-endoperoxide synthase 2) and cytosolic prostaglandin E2 synthase in RAW 264.7 cells.^48^

## 4 Antimicrobial Activity Assays

The antimicrobial activities of pure and Au-doped ZnO NCs were investigated against different strains, shown in Table 2. Au-doped ZnO NCs presented the lowest minimum inhibitory concentration (MIC) values (7.80 μg/mL) against strains of *S. aureus* and *E. coli*, except ZnO:10Au, because these NPs require higher concentrations to determine the inhibitory effect. The highest MIC concentrations were obtained for the *E. coli* species, with the best performance observed in tests with ZnO:5Au (62.50 μg/mL).

**Table 2:** Minimum Inhibitory Concentration (MIC) (μg/mL) of Pure and Au-Doped ZnO NCs and the Positive Control Meropenem^a^

	nanoproducts (μg/mL)				
bacteria species	ZnO	ZnO/0.5Au	ZnO/5.0Au	ZnO/10.0Au	meropenem
*S. aureus*	7.80	7.80	7.80	15.60	1.95
*E. coli*	125.00	125.00	62.50	250.00	
*P. aeruginosa*	7.80	7.80	7.80	31.20	

a ZnO, Zinc Oxide; Au, Gold; μg, micrograms; mL, mililiter; *S. aureus*, *Staphylococcus aureus*; *E. coli*, *Escherichia coli*; *P. aeruginosa*, *Pseudomonas aeruginosa*.

The MIC values for *S. aureus* and *P. aeruginosa* remained unchanged in the tests carried out for ZnO, 0.5, and 5.0Au samples, with an increase in the MIC at the highest concentration tested (ZnO:10.0Au). The MIC values obtained against *E. coli* adopted a similar behavior. However, an intermediate concentration of ZnO:0.5Au was up to four times more efficient in inhibiting bacterial growth than the other MICs obtained. There was no difference (*p* < 0.7038) between the MIC obtained against *S. aureus*, *E. coli*, and *P. aeruginosa* for ZnO, 0.5, 5.0, and 10.0 Au samples compared to standard treatment (Meropenem initial concentration 1000 μg/mL). However, the MIC values obtained for ZnO:5.0Au were like others (ZnO, ZnO:0.5Au) and lower for ZnO:10.0Au for *S. aureus* and *P. aeruginosa* and lower than all other concentrations for *E. coli*. This suggests that the ZnO:5.0Au sample may be more efficient against Gram-positive and Gram-negative bacteria and that high concentrations of Au (ZnO:10.0Au) can reduce the antimicrobial activity of Au-doped ZnO NCs. These results were also obtained by Khormali et al.^49^ when testing Dy2O3 (dysprosium (III)oxide)/ZnO–Au ternary nanocomposites, obtaining MIC and MBC values of 5.40 mg/ml against *S. aureus*.

Doping the ZnO NCs is advantageous with other ions or sinergim with other nanoparticles^50^ to perform mechanisms harmful to cellular metabolism, including the production of ROS, the release of ions, and direct damage to the structural components of cells.^27^ However, as nanoparticles do not act in a specific way, more than one mechanism can simultaneously be involved in antimicrobial activity.^51^ In this study, it is believed that the Au doping ZnO NCs interfered with the physicochemical properties of this nanoproduct, hindering its mechanisms related to antimicrobial activity.

The increase of bacterial death-induced NCs may be related to the mechanism of excessive production of ROS, which is considered to be derived from the increased activation of NOX2, a mechanism of antimicrobial activity mediated by macrophages.^52^ This property was seen in ZnO: Au-based nanocrystals that increased the antimicrobial performance of the nanoproducts against bacteria of Gram-positive and Gram-negative strains.^51,52^ Similarly, the action of single-walled carbon nanotubes (SWCNTs) associated with ZnO–Au hybrid nanoparticles increases the bactericidal activity of phagocytic cells against *E. coli*.^52^

ZnO NPs, obtained through different chemical and green synthesis routes, have been widely applied in antimicrobial perspectives, showing promising performance against both Gram-positive and Gram-negative bacteria, although limitations regarding their spectrum of action have been observed.^53−55^ In this context, the combination with other metallic nanoparticles, such as ZnO/AgNPs and Co–ZnO NPs, has been a strategy used to enhance the antimicrobial effect of the nanoproduct.^56,57^ Although this study did not directly compare nanoproducts obtained from the incorporation of other metallic nanoparticles, recent studies present results similar to ours, reporting an improvement in antimicrobial activity with the incorporation of the nanoparticles. Furthermore, there is evidence that the incorporation of Au NPs exhibits better antimicrobial performance compared to the Ag/ZnO NP combination.^58,59^ Doping of ZnO with metallic nanoparticles such as Ag has also been shown to be effective in controlling resistant bacteria.^58,60^ Furthermore, they have shown efficacy in controlling parasites such as tegumentary Leishmaniasis.^61,62^

### 3.4 Au-Doped ZnO Nanocrystals Exhibit Higher Biocompatibility in Fruit Flies

**Figure 3:**
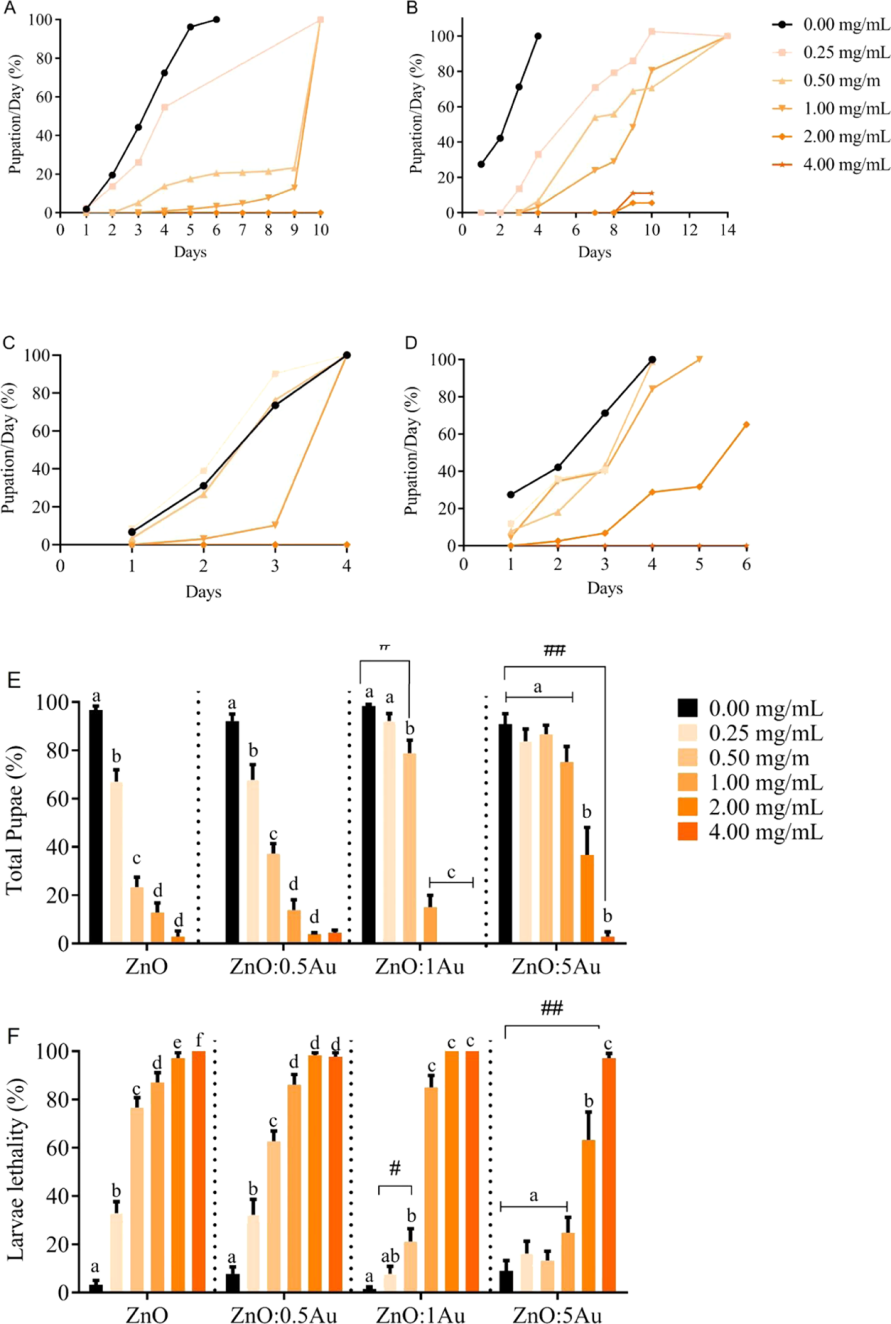
Changes in the postembryonic development of *D. melanogaster* in standard culture medium supplemented with pure and Au-doped ZnO (0.5, 1.0, and 5.0 Au). Pupation rate (%) from the negative control group and after exposure to ZnO nanocrystals (A) and 0.5Au (ZnO:0.5Au) (B), 1.0 Au (ZnO:1Au) (C), and 5.0 Au (ZnO:5Au) (D). (E) Percentage of total pupal formation. (F) Percentage (%) of larval lethality. Data are represented as a geometric mean. ^a,b,c^ Different letters represent existing differences between concentrations and the control of a nanocrystal. ##Represents the existing difference between nanocrystals with different doping concentrations (*p* < 0.0001) determined by the two-way ANOVA test followed by the Tukey mean test. Values are shown as mean ± standard error of mean (*n* = 6; 35 larvae per replicate).

Although ZnO NCs are highly toxic after *in vivo* exposure, we observed that larvae exposed to ZnO/Au showed an increase in the rate of daily pupal formation as Au doping increased. The presence of ZnO:0.5Au NCs in the standard culture medium caused a 2–4 d delay for animals exposed to concentrations from 0.25 to 1.0 mg/mL (Figure 3B). On the other hand, animals exposed to ZnO:1.0Au showed a delay of only 1 day and only at a concentration of 1 mg/mL (Figure 3C). However, when larvae were exposed to ZnO:5.0Au, there was no delay in forming pupae in animals exposed from the lowest (0.25 mg/mL) concentration up to 1.0 mg/mL (Figure 3D). Therefore, ZnO nanocrystals (NCs) doped with 1.0 and 5.0 Au demonstrated higher biocompatibility compared to pure ZnO at concentrations of 0.25, 0.50, and 1.0 mg/mL. Similar delays in postembryonic development have also been reported in other studies where larvae were exposed to varying concentrations of ZnO NCs,^53^ metal NPs,^54^ and TiO_2_ in the anatase phase.^34^ Larvae can ingest approximately 3 microliters of food per day (almost twice their weight) to compensate for the energy spent in the development process and to accumulate energy for the metamorphosis process.^55^ Therefore, the toxicity observed at this stage of larval development can be highly relevant since animals accumulate large amounts of NCs.

In the present study, we tested the biocompatibility of ZnO:0.5, ZnO:1.0, and ZnO:5.0Au NCs using *D. melanogaster* as a model. As expected, exposure to ZnO NCs caused a delay of 2 days for the onset of pupal formation, starting from the lowest concentration (0.25 mg/mL). On the other hand, animals exposed to 1 mg/mL showed a lower pupa formation rate; for the highest concentrations (2.0 and 4.0 mg/mL), we observed practically no pupa formation (Figure 3E). This result, coupled with the low total pupal formation rate (Figure 3E) and high larval lethality (Figure 3F), highlights the significant toxicity induced by ZnO nanocrystals (NCs) in living organisms. It is noteworthy that both pupal and larval mortality were concentration-dependent (Figure 3E,F).

There was a highly significant relationship between larval mortality (*p* < 0.0001) and the total percentage of pupae formed (*p* < 0.0001) both among concentrations and among different samples of Au-doped ZnO. ZnO and ZnO:0.5Au NCs were toxic to larvae in a concentration-dependent manner. We observed high rates of larval lethality (Figure 3F) and low total pupation rates (Figure 3E), which consequently resulted in few emerging adults. Surprisingly, there was a reduction in the larval lethality rate and an increase in the pupal formation rate in the animals grown in standard culture medium supplemented with ZnO:1.0Au at 0.25 and 0.50 mg/mL or with ZnO:5.0Au at 0.25–1.0 mg/mL. The rates observed were similar to the control and differed significantly from those found for animals exposed to ZnO and ZnO:0.5Au nanocrystals (*p* < 0.0001). This result demonstrates that increased Au doping leads to improved biocompatibility of the nanocrystals (NCs), as evidenced by reduced mortality, and corroborates the findings of the *in vitro* assays conducted in this study.

We found that the toxicity of NCs is concentration-dependent, and concentrations above 1 mg/mL are highly toxic for pure and Au-doped ZnO NCs at any concentration of Au. In the highest concentrations evaluated (2.0 and 4.0 mg/mL), we observed low daily pupal formation (Figure 3), high larval mortality rate, and low pupal formation (Figure 3) for the exposure of animals to all NCs. It is already established that ZnO nanoparticles (NPs) are toxic to Drosophila and can induce the increased production of reactive oxygen species (ROS), resulting in oxidative stress and genotoxicity.^63−66^ The high generation of ROS may be the cause of both the delay in postembryonic development and the high larval lethality after exposure to ZnO and ZnO:0.5Au [51,53,54]. ZnO NPs cause toxicity because they induce the generation of excess ROS and disrupt the flow of Ca^2+^, leading to cellular distress, which consequently induces a series of cellular damage, such as changes in Nrf2 signaling pathways, reduction of antioxidant proteins such as glutathione (GSH),^58^ redox imbalance, and mitochondrial dysfunction, which in the end lead to cell death.^17,54^

We suggest that possible mitochondrial dysfunctions and reduced levels of GSH synthesis are the causes of the alterations in larval development found in this study. This is because ecdysone biosynthesis depends on GSH, and part of it occurs in mitochondrial cytochrome P450. This hormone is the crucial molecule for initiating pupal formation, so a delay or defect in releasing ecdysone can delay larval development.^59,66,67^ Another essential factor is that ZnO reduces the expression of essential marker proteins in the Wnt signaling pathways. These proteins determine various stages of development, and their reduction can also result in developmental defects in fruit flies.^68^

**Figure 4:**
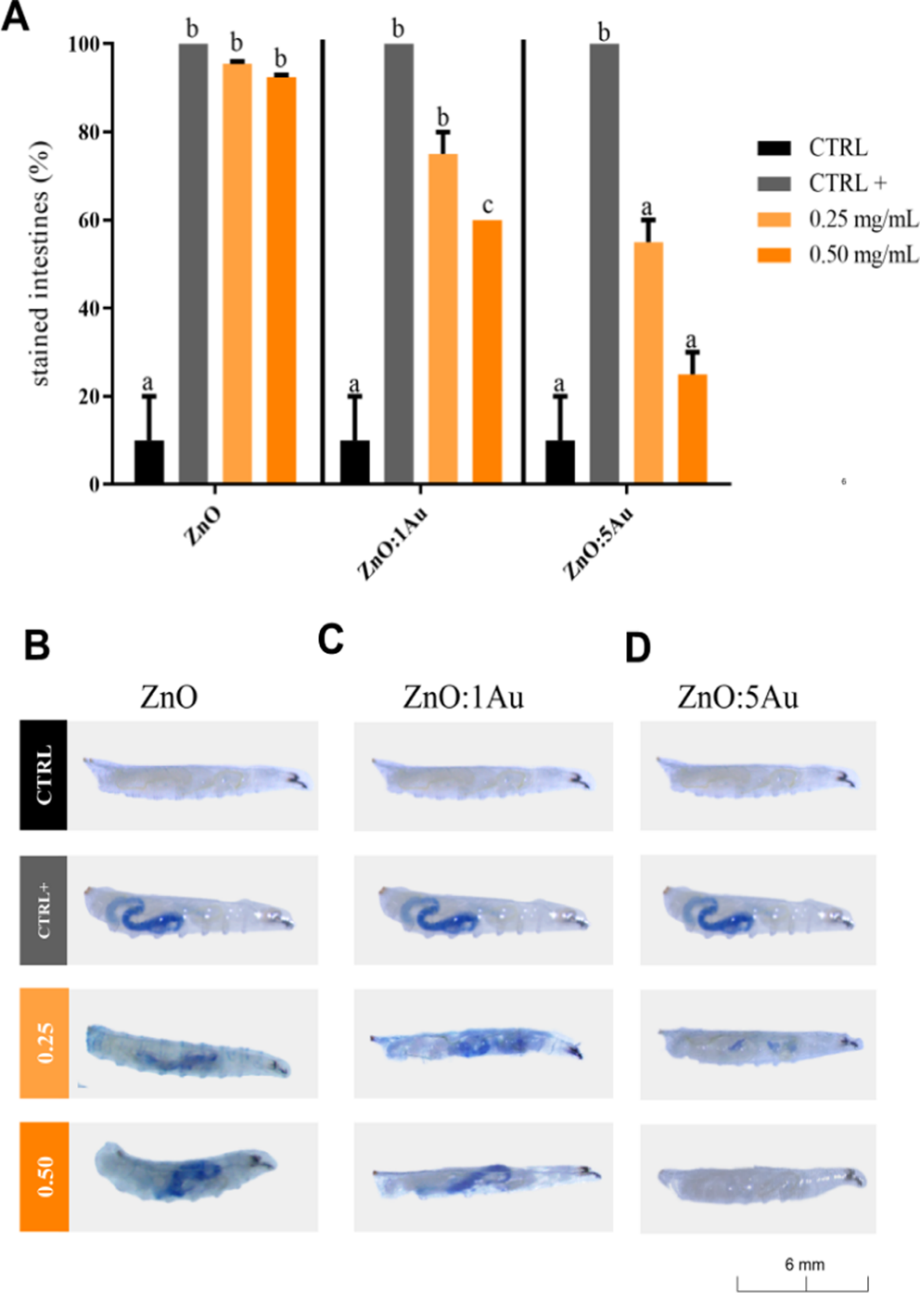
Cytotoxicity analysis in the intestines of *D. melanogaster* larvae (L3) exposed orally to nanocrystals of pure ZnO and Au-doped ZnO. (A) Percentage of animals exhibiting stained intestines following exposure to ZnO and Au-doped ZnO (1.0 or 5.0) Au at concentrations of 0.25 and 0.50 mg/mL, respectively. ^a,b,c^Different letters represent existing differences between nanocrystal concentrations and the control group. (B) Representative images of orally exposed larvae (L3) to nanocrystals containing ZnO. (C) Representative images of exposed larvae (L3) to ZnO nanocrystals containing 1.0 Au. (D) Representative images of exposed larvae (L3) to ZnO nanocrystals containing 5.0 Au. The blue coloration, indicating affected areas, was observed in the clusters of intestinal cells.

We observed that doping ZnO with 1.0 and 5.0 of Au increased the biocompatibility. These results can be supported when we observe the low larval lethality and high rate of pupal formation (Figure 3) in the groups exposed to ZnO:1.0Au and ZnO:5.0Au (at concentrations below 1 mg/mL). To reinforce these data, we also observed lower cytotoxicity in intestinal cells of larvae (L3) when they were exposed to Zn:1.0Au or ZnO:5.0Au at a concentration of 0.50 mg/mL when compared with a concentration of 0.25 mg/mL and to the control (Figure 4 and Supporting 1). We even found that ZnO:5.0Au at a concentration of 0.5 mg/mL did not show cytotoxicity (Figure 4D) because the exposed group showed the same percentage of stained larvae in blue as the control. (A portion of the larval intestine was also examined at 45× magnification using a stereoscopic microscope, Supporting 1.)

The pathway by which doped NCs cause toxicity may be different from that suggested for ZnO since we did not observe a high rate of ROS formation in the chemical oxidation assays of this study, and the formation of NO in cells exposed to ZnO doped with 1.0 or 5.0 Au was low. This possibly occurred due to the new structure of NCs, where Zn^2+^ ions are replaced by Au, suggesting a low release of Zn^2+^ ions and possibly leading to less ROS formation. This would also explain why high-Au-doped NCs do not show good bactericidal activity. Therefore, we suggest that doping with Au reduces the formation of ROS and thus becomes more biocompatible, and consequently, there is less activation of cell death signaling pathways. This can also be supported by our degradation results, which demonstrate that the higher the Au doping, the lower the photodegradation and consequently lower ROS formation.

**Figure 5:**
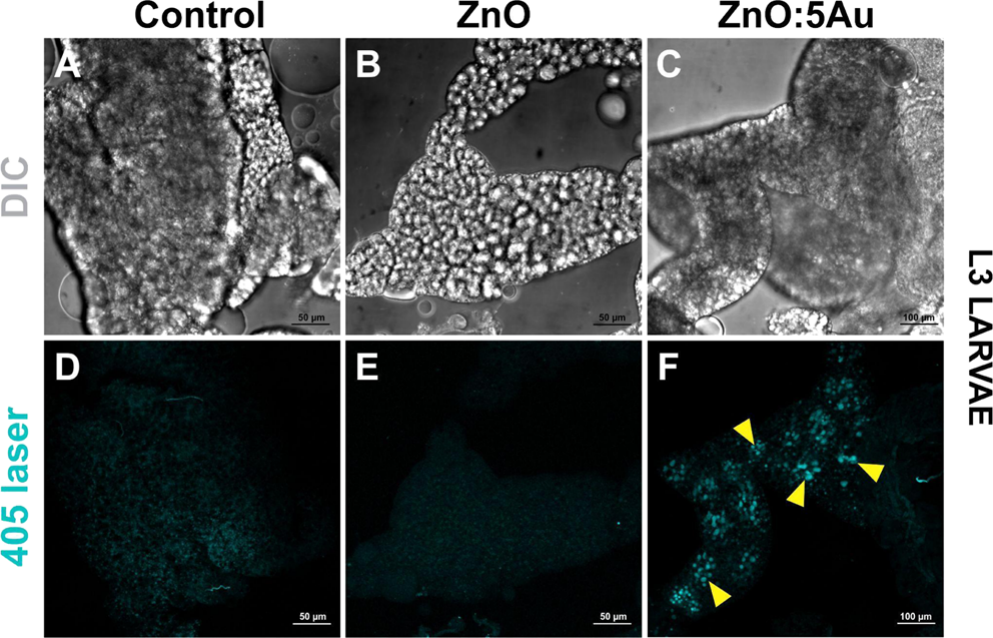
Cellular distribution of ZnO:5Au NCs in the third instar larval fat body of *Drosophila* by confocal microscopy. (A, D) Fat body of unexposed third instar larvae fed in standard food medium showing any signal of nanocrystal luminescence. (B, E) The fat body of third instar larvae exposed to 1 mg/mL of ZnO also shows no discernible luminescence. (C, F) Nanocrystals aggregate in the fat body tissue of third instar larvae exposed to 1 mg/mL of ZnO:5Au, as indicated by yellow arrowheads. DIC: differential interference contrast; cyan: 405 nm laser used for nanocrystal luminescence excitation.

**Figure 6:**
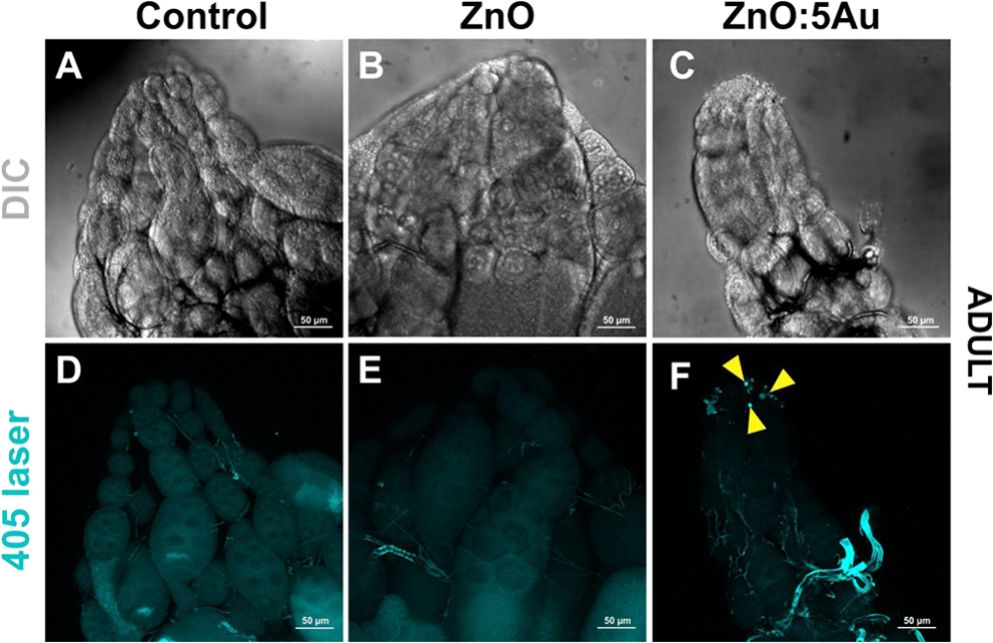
Cellular distribution of ZnO:5Au nanocrystals in the *Drosophila* ovary of adult females by confocal microscopy. (A, D) Ovaries dissected from adult females fed in standard food medium show any signal of nanocrystal luminescence. (B, E) Adult females exposed to 1 mg/mL of ZnO also show no discernible luminescence in the ovaries. (C, F) Nanocrystals aggregate in muscle sheath cells in the anterior region of the ovaries of adult females exposed to 1 mg/mL of ZnO:5Au, as indicated by yellow arrowheads. DIC: differential interference contrast; cyan: 405 nm laser used for nanocrystal luminescence excitation.

**Figure 7:**
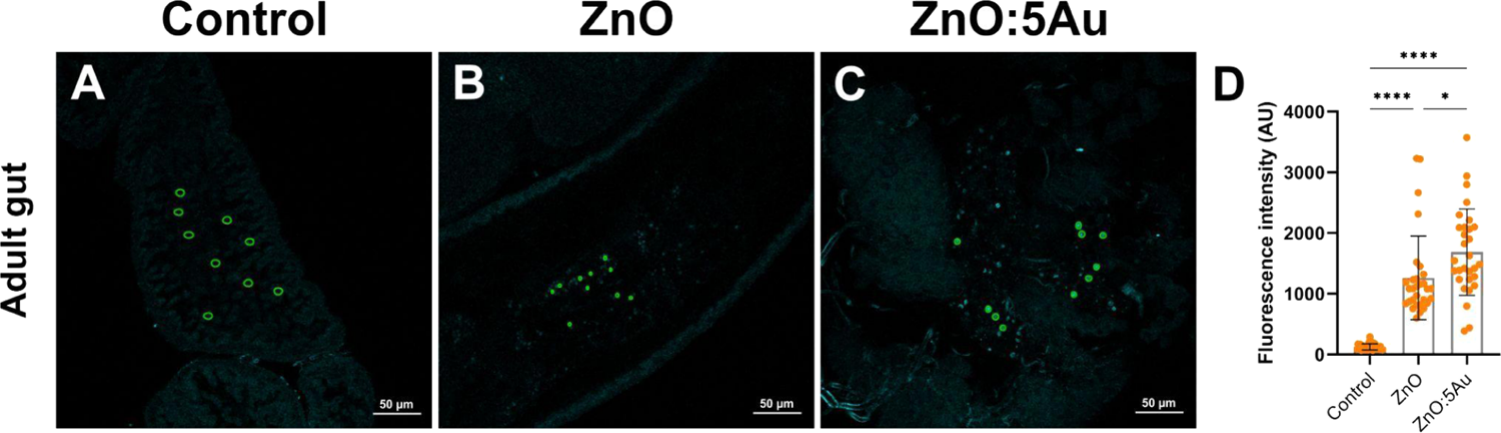
Luminescence intensity quantification of ZnO and ZnO:5Au NCs dissected in the *Drosophila* adult gut. (A–C) Representative confocal images of the adult gut were used in the analysis. Green circles indicate ROIs applied to quantify the luminescence intensity. (D) Analysis of variances (ANOVA) of luminescence intensity using 30 ROIs in different confocal images of *Drosophila* adult gut showing the higher luminescence signal of ZnO:5Au compared to the control and ZnO NCs. The quantification was carried out through the ROI statistics plugin on the Icy software (Chaumont et al.^41^).

**Figure 8:**
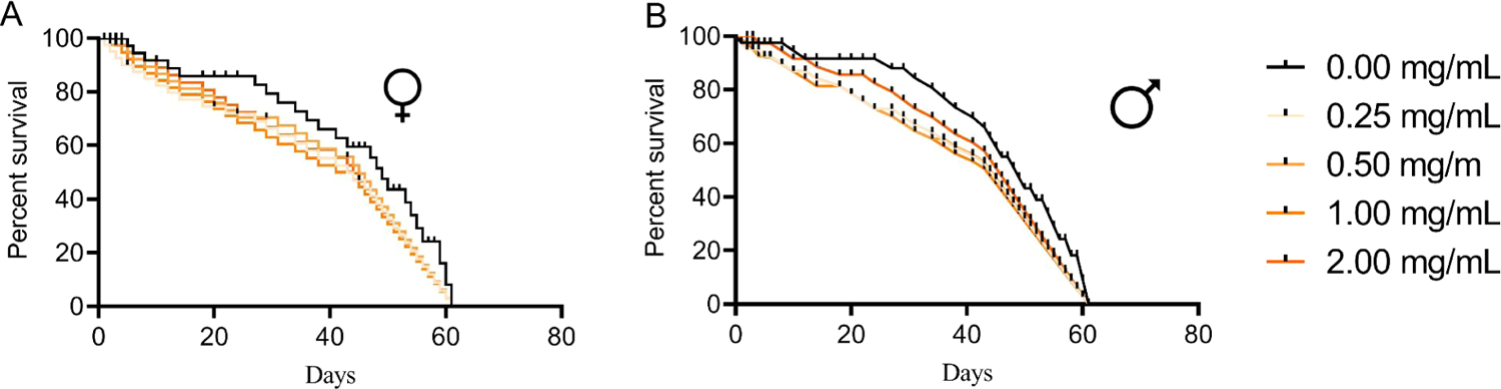
Percent survival of (A) females and (B) males of the *D. melanogaster* after chronic exposure during the larval stage of ZnO:5Au.

As ZnO:5Au was the most biocompatible NCs, we investigated whether it would be possible to trace them in larvae (L3) and adults of *D. melanogaster*. Interestingly, we observed the luminescence of ZnO:5Au nanoparticles within the intestine of both larvae and adults of fruit flies (Supporting 2). Furthermore, upon quantifying the luminescence intensity, we discovered that Au-doped ZnO exhibited a higher intensity compared to that of pure ZnO NCs at the same concentration (Figure 7). Additionally, we observed luminescence from NCs in the ovaries of adults (Figure 6) and in the fat body of L3 larvae (Figure 5). As mentioned above, larvae consume a large amount of food, so we expected to find NCs in the fat body. Similar results were found for larvae exposed to TiO_2_^60,63^ and Eu-doped TiO_2_.^35^ We know that after the metamorphosis process, the accumulation of NCs in the fat body is secreted, likely without toxic changes for adult animals.^35^ Corroborating the existing literature, we also did not observe any alterations in the overall lifespan of the animals that were raised in a medium containing ZnO:5Au (Figure 8). This could indicate that Au-doped ZnO did not have an impact on the animals’ lifespan.

We have not observed cytotoxicity in the larval exposure to Au-doped ZnO at lower concentrations tested in this study (0.25 and 0.50 mg/mL). Figure 4 illustrates the potential induction of cytotoxicity by ZnO in the intestinal cells of Drosophila (Figure 4A,B), whereas larvae exposed to 1.0Au exhibited reduced cytotoxicity. However, larvae exposure at 5.0Au showed a drastic blue staining in the animals’ intestines, indicating greater biocompatibility about ZnO and 1.0Au. These results suggest that there may be minimal cellular damage that is potentially not significant enough to significantly impact cellular viability.

This study not only verified the exposure of animals to the NCs but also unveiled a novel property of nanoparticles. This newly identified attribute suggests a high potential for the application of these ZnO:5.0Au in biomedicine, specifically in bioimaging and drug delivery tracking, in addition to their bactericidal properties. Consequently, Au-doped ZnO NCs emerge as promising biocompatible nanocrystals with theranostic capabilities.

The results of our investigation underscore the significance of nanomaterial doping techniques, demonstrating that such modifications not only enhance the physicochemical properties of the nanocrystals but also contribute to their improved biocompatibility. This finding has implications for the development of advanced nanomaterials with multifunctional applications in therapeutic and diagnostic domains.

It is crucial to underscore the limitations of this study, particularly with regard to elucidating the specific cellular and molecular pathways responsible for the observed cytotoxicity. Future investigations should delve into exploring hormonal levels, quantifying additional antioxidant enzymes, and assessing tissue reactive oxygen species (ROS) levels. A comprehensive understanding of these mechanisms is imperative for gaining insights into the intricate interactions between nanocrystals and biological systems. It is noteworthy that, in this study, long-term exposure to Drosophila was precluded, leading to a focus on short-term analyses during postembryonic development. However, acknowledging the necessity for long-term analyses and adult bioassays is essential to elucidate the persistence and potential aggregation/agglomeration of nanocrystals over time and, thereby, assess their safety and efficacy comprehensively.

The antimicrobial activity assays, while informative, are subject to certain limitations. A more in-depth, time-dependent study could provide valuable insights into the kinetics of microbial inhibition, shedding light on whether the nanocrystals sustain their effectiveness over prolonged exposure periods. The study did not extensively explore the specific mechanisms underpinning the nanocrystals’ antimicrobial effects. Enhancing our understanding of whether these effects result from reactive oxygen species (ROS) generation, membrane disruption, or other mechanisms would significantly advance the mechanistic comprehension of their antimicrobial properties.

Furthermore, in characterizing nanocrystals, it is imperative to acknowledge certain limitations in the methodology employed. Addressing these limitations ensures a more nuanced interpretation of the results and promotes a more robust foundation for future research in this domain.

## 5 Conclusions

We synthesized and confirmed the formation of pure and Au-doped ZnO NCs. The doping with Au does change the phase and size of ZnO NCs, but introducing a high level of Au ions into the ZnO NCs decreases the photocatalytic activity. Cells exposed to these nanocrystals demonstrated cytotoxicity that depended on the concentration. However, when exposed to nanocrystals doped with 1.0 and 5.0 Au, the cells exhibited low NO formation, suggesting potential nontoxicity. Additionally, in our experiments using the well-established *D. melanogaster* nanotoxicity model, we observed that ZnO nanocrystals with 1.0 and 5.0 of gold doping were less toxic at concentrations below 1.0 mg/mL. In microbiological tests, ZnO:5Au showed the most favorable outcome by reducing the MIC of *E. coli* (62.5 μg), without affecting the MIC of *S. aureus* and *P. aeruginosa*. Therefore, it is suggested that ZnO:5Au is the most biocompatible sample at concentrations below 1.0 mg/mL. However, further investigations are required to determine the mechanisms responsible for its enhanced biocompatibility compared with other NCs.

## Supporting Information Available

Cytotoxicity in intestinal cells of *Drosophila melanogaster*; a portion of the larval intestine was examined at 45× magnification using a stereoscopic microscope (PDF)
