# Supplementary material for: Tuning Biocompatibility and Bactericidal Efficacy as a Function of Doping of Gold in ZnO Nanocrystals
Source: ACS Omega. 2024 May 10;9(20):21904–16. doi: 10.1021/acsomega.3c09680 (PMC11112696; doi:10.1021/acsomega.3c09680)
Supplement: Supplementary file 1 — ao3c09680_si_001.pdf [file ao3c09680_si_001.pdf]

Supplementary Material

Tuning Biocompatibility and Bactericidal Efficacy as a Function of Doping of Gold in ZnO Nanocrystals

Jerusa Maria de Oliveira[1,3]†, Davi P. da Silva[1,2,4]†, Luciana Rosa de S. Floresta[1,3]†, Gustavo G. Rocha[1,5], Larissa Iolanda Moreira de Almeida[1,3], Edigar Henrique V. Dias[5], Thaís Karine de Lima[6], Juliane Z. Marinho[6], Marylu M. de Lima[8], Felipe B. Valer[8], Fábio de Oliveira[9], Thiago L. Rocha[7], Valter Alvino[4], Lucas Anhezini[3]†, Anielle Christine A. Silva[1,2]**

[1] Strategic Materials Laboratory, Physics Institute, Federal University of Alagoas, Maceió, CEP: 57072-900 Alagoas Brazil.
[2] Rede Nordeste de Biotecnologia (RENORBIO), Chemistry Institute, Federal University of Alagoas, Maceió 57072-900, Alagoas, Brazil
[3] Laboratory of in vivo Toxicity Analysis, Institute of Biological Sciences and Health, Federal University of Alagoas, Maceió, Alagoas, Brazil
[4] Laboratory of Wound Treatment Research, Institute of Pharmaceutical Sciences, Federal University of Alagoas, Maceió, Alagoas, Brazil
[5] Department of Medicine, Biotechnology Institute, Federal University of Catalão, Catalão, Goiás, Brazil.
[6] Institute of Chemistry, Federal University of Uberlândia, Uberlândia, Minas Gerais, Brazil.
[7] Laboratory of Environmental Biotechnology and Ecotoxicology, Institute of Tropical Pathology and Public Health, Federal University of Goiás, Goiânia, Goiás, Brazil.
[8] Department of BioMolecular Sciences, School of Pharmaceutical Sciences of Ribeirão Preto, University of São Paulo, Ribeirão Preto, São Paulo, Brazil.
[9] Laboratory of Molecular and Cellular Biology, Institute of Biomedical Sciences, Federal University of Uberlândia, Uberlândia, Minas Gerais, Brazil.

† These authors contributed equally to this work.

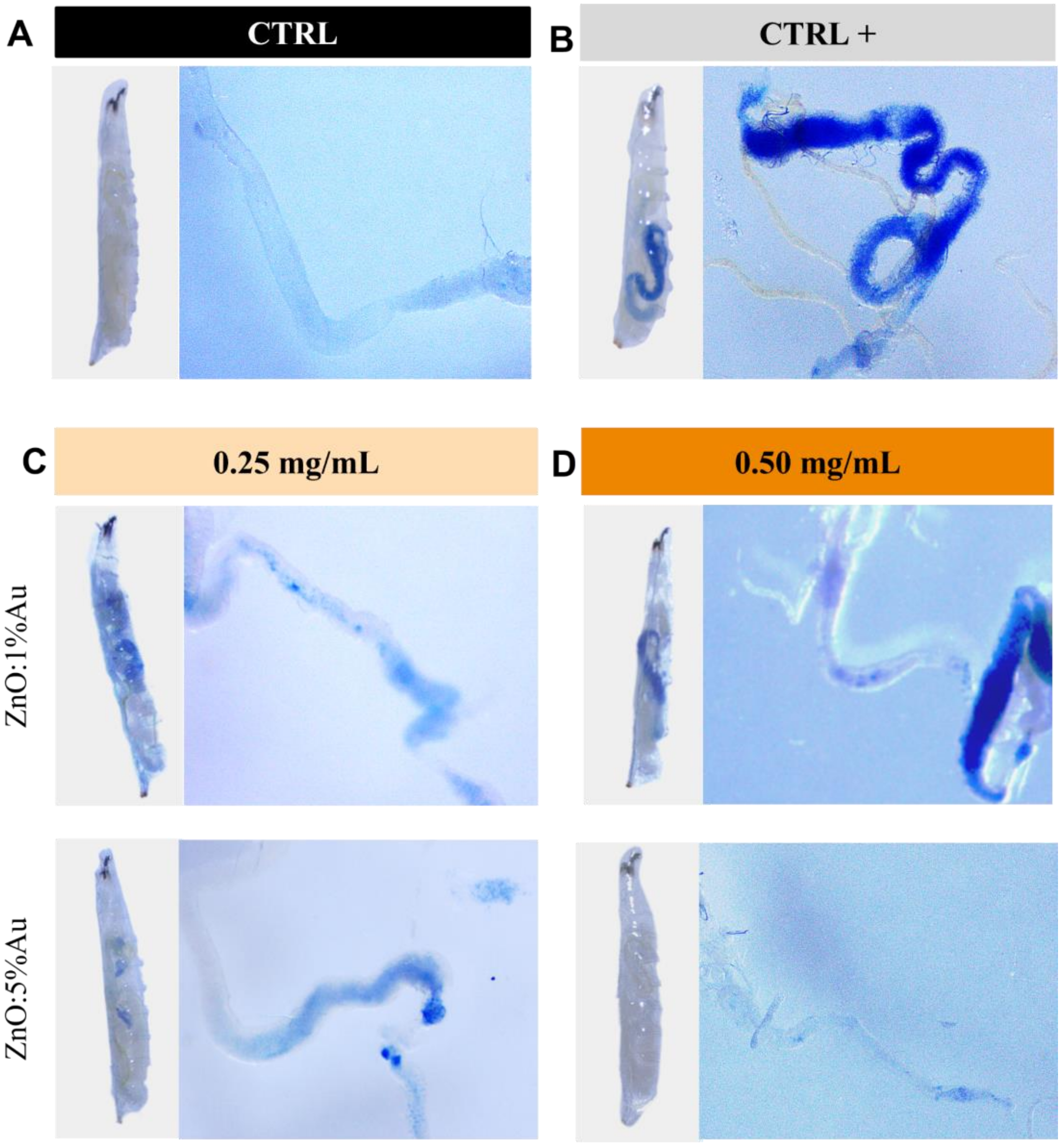

Supplementary 1: Cytotoxicity in intestinal cells of *Drosophila melanogaster* was observed when exposed to pure ZnO nanoparticles (NCs) and ZnO nanoparticles doped with gold (Au) at concentrations of 1% (ZnO:1.0Au) and 5% (ZnO:5.0Au) during post-embryonic development. Third-stage larvae (L3) were exposed to 0.25 and 0.50 mg/mL of pure or Au-doped- ZnO NPs. Photos of larval bodies were taken under 15X magnification using a stereoscopic microscope after performing a trypan blue dye exclusion test. The blue coloration, indicating affected areas, was observed in clusters of intestinal cells. A portion of the larval intestine was examined at 45X magnification using a stereoscopic microscope. (A) larvae control; (B) larvae exposure to a positive control (0.2 mg/mL Imazapyr ($C_{13}H_{15}N_3O_3$) (Matt Tiririca Imazapyr commercial solution, Kelldrin, 2.0% w/w).

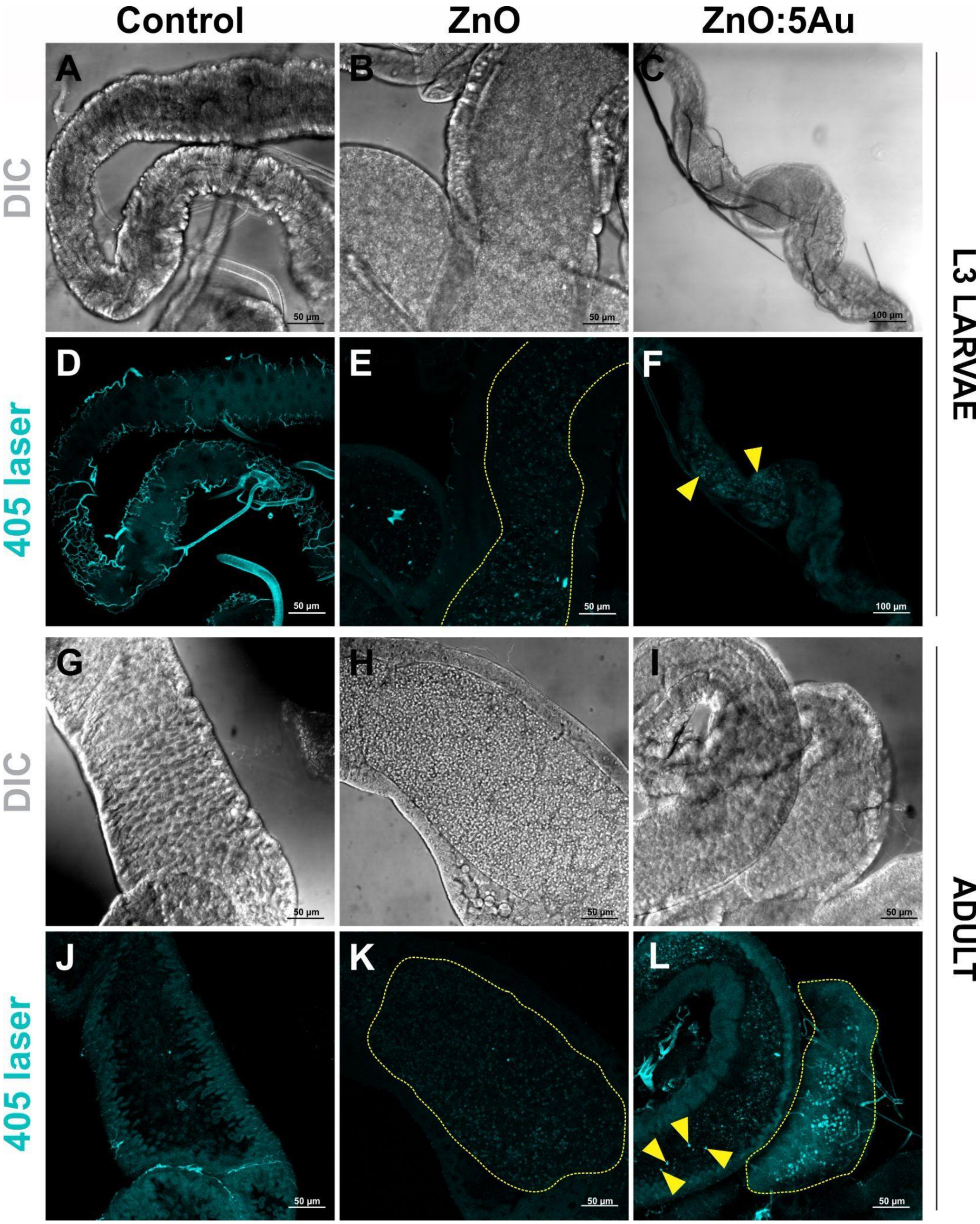

Supplementary 2. Confocal microscopy showing Zinc Oxide (ZnO) and 5% gold-doped Zinc Oxide (ZnO:5Au) nanocrystals in the larval and adult gut of *Drosophila*. (A, D) Gut dissected from third instar larvae and (G, J) adult females fed in standard food medium show any signal of nanocrystal luminescence. (B, E) Third instar larvae and (H, K) adult

females exposed to 1 mg/mL of ZnO show nanocrystals aggregate dispersed along the lumen of the gut (dashed line). (C, F) Gut dissected from third instar larvae and (I, L) adult females exposed to 1 mg/mL of ZnO:5Au show nanocrystals aggregate in the lumen of the gut, as indicated by dashed line and yellow arrowheads. DIC: differential interference contrast; Cyan: 405 nm laser used for nanocrystal luminescence excitation.
